# Using microarrays to identify positional candidate genes for QTL: the case study of ACTH response in pigs

**DOI:** 10.1186/1753-6561-3-S4-S14

**Published:** 2009-07-16

**Authors:** Vincent Jouffe, Suzanne Rowe, Laurence Liaubet, Bart Buitenhuis, Henrik Hornshøj, Magali SanCristobal, Pierre Mormède, DJ de Koning

**Affiliations:** 1 Laboratoire PsyNuGen, INRA UMR1286, CNRS UMR5226, Université de Bordeaux 2, 146 rue Léo-Saignat, F-33076 Bordeaux, France; 2 The Roslin Institute and R(D)SVS, University of Edinburgh, Roslin EH25 9PS, UK; 3 Laboratoire de Génétique Cellulaire, INRA UMR444, F-31326 Castanet-Tolosan, France; 4 Department of Genetics and Biotechnology, Faculty of Agricultural Sciences, Aarhus University, DK-8830 Tjele, Denmark

## Abstract

**Background:**

Microarray studies can supplement QTL studies by suggesting potential candidate genes in the QTL regions, which by themselves are too large to provide a limited selection of candidate genes. Here we provide a case study where we explore ways to integrate QTL data and microarray data for the pig, which has only a partial genome sequence. We outline various procedures to localize differentially expressed genes on the pig genome and link this with information on published QTL. The starting point is a set of 237 differentially expressed cDNA clones in adrenal tissue from two pig breeds, before and after treatment with adrenocorticotropic hormone (ACTH).

**Results:**

Different approaches to localize the differentially expressed (DE) genes to the pig genome showed different levels of success and a clear lack of concordance for some genes between the various approaches. For a focused analysis on 12 genes, overlapping QTL from the public domain were presented. Also, differentially expressed genes underlying QTL for ACTH response were described. Using the latest version of the draft sequence, the differentially expressed genes were mapped to the pig genome. This enabled co-location of DE genes and previously studied QTL regions, but the draft genome sequence is still incomplete and will contain many errors. A further step to explore links between DE genes and QTL at the pathway level was largely unsuccessful due to the lack of annotation of the pig genome. This could be improved by further comparative mapping analyses but this would be time consuming.

**Conclusion:**

This paper provides a case study for the integration of QTL data and microarray data for a species with limited genome sequence information and annotation. The results illustrate the challenges that must be addressed but also provide a roadmap for future work that is applicable to other non-model species.

## Background

There is a wealth of information that has been collated from many QTL studies over the last decade, summarised in resources such as animalQTLdb [1]. With the advent of 'omics technologies such as gene expression microarrays, new ways of studying genetic variation through so called 'endo-phenotypes' are becoming increasingly popular. As the volume of data from these new technologies increases, the current challenge is to combine existing knowledge gained from linkage studies with new information to propose candidate genes in QTL regions. Such an integration has previously been proposed by Fisher *et al *(2007) dealing with mouse models for trypanotolerance [2].

**Figure 1:**
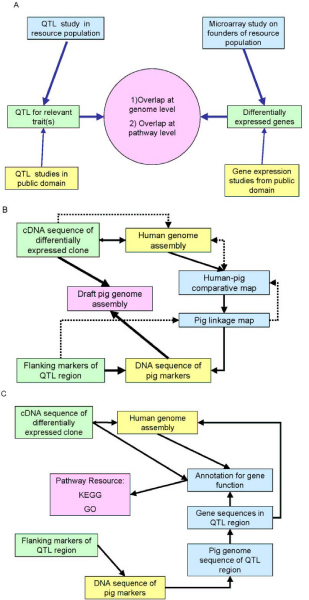
**The process of combining QTL and gene expression results**. Panel A shows the two areas of comparing gene expression results with QTL results: 1) co-location at the genome level between differentially expressed genes and QTL, 2) significant overlap at the pathway level between genes in the QTL region and differentially expressed genes elsewhere in the genome. Panel B illustrates the steps to test for co-location between differentially expressed genes and QTL regions in the pig example. The solid arrows indicate the comparisons at the level of the pig genome. The dashed arrows outline an alternative strategy where comparisons are made on the human genome, using comparative mapping approaches. Panel C illustrates the steps that are required to test for significant enrichment at the pathway level between differentially expressed genes and genes in the QTL region.

The general principle of complementing QTL studies with gene expression data is illustrated in Figure 1A. The goal is to identify overlap between differentially expressed genes and QTL regions at either the genome or the pathway level. Differentially expressed genes in the QTL region are obvious positional candidate genes for the QTL. Differentially expressed genes elsewhere in the genome might share pathways with genes in the QTL region and reflect downstream effects of the QTL. The study of genes at the pathway level is also important to identify positional candidate genes in the QTL region that are not represented on the microarray.

Linking gene expression results with QTL data has mainly been applied to studies where gene expression was measured on individuals that have a direct link to the QTL study (e.g. the founders of an F2 QTL experiment [2,3]). The principle can be extended to include QTL and gene expression results from the public domain but requires careful consideration of the biological relevance between different studies.

The goal of this paper is to present a case study to integrate QTL data and microarray data for an experiment in pig, which has only a partial genome sequence. We outline various procedures to localize differentially expressed genes on the pig genome and link this with information on published QTL.

Linking QTL and microarray results becomes quite challenging when the genome of the target species has not been fully sequenced as annotation becomes extremely dependent on identification of homologues. Figure 1B illustrates the different routes that can, or must, be taken to test for co-location at the genome level for the present study. In order to place genes on the pig genome, one might have to go via the human genome using comparative mapping approaches.

The challenges increase for comparisons at the pathway level because in order to map differentially expressed clones to pathways they need to have functional annotation (Figure 1C). Furthermore, the pathway databases are almost exclusively populated with data derived from other species than the pig.

For this part of the EADGENE/SABRE workshop, the microarray results by Hazard et al (2008) [4] were distributed among participants. The starting point for each group was a list of 237 differentially expressed transcripts. This article summarises the analyses of three different groups that analysed the data independently: the analyses performed by VJ, LL, MSC and PM will be indicated by INRA; the analyses by BB and HH are indicated by AARHUS while those from SR and DJK are denoted ROSLIN. Where appropriate, we have integrated results into single tables and figures while some of the original results from the separate groups are provided as supplementary files (Additional file 1 and 2). In line with the other workshop papers, this manuscript will focus on the results of the various analyses and, in doing so illustrate the opportunities and challenges of combining microarrays and QTL. This paper provides several strategies as well as actual scripts (Additional file 3) for integration of QTL and gene expression results that are portable to other species.

## Methods

Hazard *et al. *(2008) [4] compared gene expression levels in the adrenal glands of Large White and Meishan pigs in basal state and after treatment with adrenocorticotropic hormone (ACTH). They report 241 differentially expressed transcripts with FDR < 0.05, representing 211 unique genes and 25 unknown transcripts. For 108 genes with P < 0.0001, 51 are differentially expressed for the genotype, 21 are differentially expressed for ACTH treatment and 36 were significant for both genotype and treatment. Although the latter are most promising in relation to QTL for ACTH response, no distinction was made between these genes for the post analyses workshop.

Désautés *et al. *(2002) [5] looked at QTL regions associated with basal levels and stress responses by studying behaviour, ACTH and cortisol levels after exposure to a stressful environment. Furthermore, cortisol has potent metabolic effects and influences numerous traits related to production, such as growth rate and carcass composition. Therefore, it is of interest to examine whether genes differentially expressed in the adrenal gland could be mapped to published QTL related to production traits. Finally, we used this mapping information to explore the underlying functional pathways and mechanisms involved in the differences between the two breeds.

### Analyses by the INRA group

Two types of analyses were performed: 1) a focussed analysed of 12 genes, 2) a global analyses for co-location between all differentially expressed genes and QTL from the public domain.

### Focussed analyses of 12 genes

Among the differentially expressed genes from Hazard et al. (2008) [4], the nine most differentially expressed genes (FDR<10^-6^) that have been functionally annotated have been studied in more detail: the ring finger protein 2 (Rnf2, BX673517), the Cbp/p300-interacting transactivator, with Glu/Asp-rich carboxy-terminal domain, 1 (Cited1, BX918031), the cAMP responsible element modulator (Crem, BX670994), the CD83 molecule (Cd83, AW42951), the eukaryotic translation initiation factor 1B (Eif11b, BX926052), the alanyl aminopeptidase (Anpep, BX665286), the acyl-Coenzyme A oxidase 1, palmitoyl (Acox1, BX673098), the alpha-2-macroglobulin (A2m, BX674240), the growth arrest and DNA-damage-inducible beta (Gadd45b, BX671980) genes. The steroidogenic acute regulator (Star, BX669487), the low density lipoprotein receptor (Ldlr, BX673438) and the creatine kinase, brain (Ckb, BX920566) have been added because their role into the adrenal sensitivity to adrenocorticotropic hormone is known.

The sequences have been downloaded from the SIGENAE website . These twelve genes were first aligned to the human genome on ENSEMBL release 49 and 50 (for the Cd83 and Rnf2 genes) on ENSEMBL website  using BLAT program [6] with default parameters.

Subsequently, these genes have been mapped on pig chromosomes by synteny using the comparative map [7]. Finally, these genes were also localised on the draft pig genome sequence (release 6) that was downloaded from the ftp Sanger website: . This release of the draft genome sequence was incomplete and no sequence data for chromosomes 3, 9, 16 and 18 was present. The sequences have been formatted for alignment using formatdb software[8]. The twelve most differentially expressed genes have been aligned to the pig chromosomes using BLASTn program [8] from the blastall tool [9] with default parameters.

In the next step, the locations of these 12 genes were compared with published QTL results. QTL data were downloaded from the Pig Quantitative Trait Locus database (PigQTLdb) [10,11]. PigQTLdb release 6 contains 1,831 QTL from 113 publications. Those QTL describe mainly meat quality traits but also health, production and reproduction traits. Genes were considered to be co-localized with QTL when they were positioned within the confidence interval of the QTL as indicated at PigQTLdb.

### Analysis of all the differentially expressed genes and QTL for stress responses

The second part of the work illustrates another approach to study differentially expressed gene in the context of relevant QTL. This approach was presented previously [12] in order to emphasize a subset of genes among a larger list of differentially expressed genes.

First, all the differentially expressed genes [4] have been systematically localized on the human genome. This was done with the BioMart software available on the Public Sigenae Contig Browser:

.

Secondly, we recovered the QTL published for analysis of behavioural and neuroendocrine responses to stress conducted in a three-generation experimental cross between Meishan and Large White pig breeds [13] from pigQTLdb [10,11]. Using the position of the QTL on the genetic map, a putative orthogonal genomic position could be obtained with a human chromosome. The comparative mapping strategy between human and pig and the several pig maps (genetic and RH) aims to identify syntenic regions.

The results from the focussed and the global analyses have been compared in order to corroborate the localization of regulated genes in QTL regions. To obtain more precise information, the candidate genes should also be mapped on the pig RH map using the INRA-Minnesota 7000 rads radiation hybrid panel (IMpRH) as was done in a previous work [12].

### Analyses by the AARHUS group

The aim was to map the differentially expressed genes to the porcine genome in order to test for overlap between differentially expressed genes and QTL. The accession numbers of the 237 differentially expressed genes were used to extract the gene sequences from the nucleotide database from NCBI. The sequences were mapped on the pre-ensemble (release 6) porcine genome using the BLASTN program. The pig genome sequence database did not contain information on SSC3, SSC9, SSC16 and SSC18.

### Analyses by the Roslin group

There were three main routes of interest: 1) accurately position differentially expressed genes in the genome to see whether they co-locate with regions of suggestive or confirmed linkage; 2) look at the genes under a QTL peak and see if they match the differentially expressed genes; 3) perhaps of greatest interest is to examine whether the genes under a QTL peak belong to the same pathway or regulatory network as differentially expressed genes and whether these pathways link QTL peaks on different chromosomes together i.e. are enriched in identified QTL regions compared to the rest of the genome.

### Positioning of differentially expressed genes

Version 7 of the porcine genome was downloaded from the Sanger website.  and was formatted into a searchable database using the software formatdb [8]. Using the GenBank accession numbers from the list of 211 differentially expressed genes, fasta files were extracted containing the sequence for each gene. Each gene sequence was blasted against pig genome using blastall [9]. A word length of 20 (-W 20) was used to reduce the number of spurious hits. We used the blast results to ascertain the most likely position of each differentially expressed gene.

### Identifying genes under the QTL peak

To identify which of the differentially expressed genes were under the QTL regions for stress responses described by Désautés et al. (2002) [14], the flanking markers of the QTL needed to be accurately placed on the pig genome. Analogous to the genes, the sequence of the flanking markers was blasted against the draft porcine genome. The QTL on chromosome 1 was flanked by S0155 and S0374, corresponding to 144–160 Mb on the draft genome sequence. The QTL on chromosome 7 was flanked by S0101 and SW2446, corresponding to 112–125 Mb on the draft genome sequence. Using Entrez gene  we obtained a list of genes that are located between these markers.

### Annotation and pathway analysis

In order to examine pathways and shared pathways gene identifiers from the NCBI gene report can be used. There are numerous sources of identifiers linking into pathway databases. The first step is to confirm the KEGG identifiers, and from these link to the KEGG pathway identifiers, and subsequently map to pathways and get the relevant GO terms. The following FTP sites were queried to obtain the relevant pathways and GO terms: , , , and 

All scripts for these procedures are available in Additional file 3.

## Results and discussion

### Focussed analysis of 12 genes (INRA)

**Figure 2:**
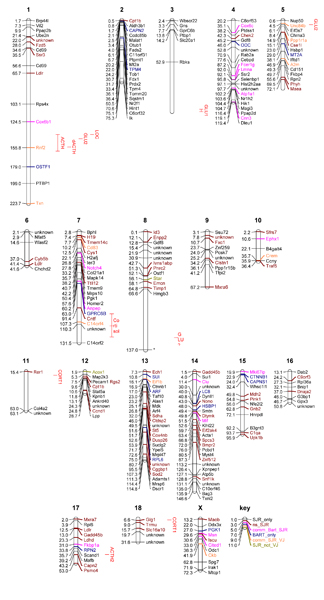
**Inferred locations of differentially expressed genes and ACTH response related QTL on the porcine genome**. Positions are given in Mb derived from the draft assembly of the pig genome (version 7). Black gene symbol denotes genes positioned by SJR using blast against pig genome sequence build 7, brown gene symbol denotes best position for a gene although not significant in the blast output (e-value < 0.0001). Pink are genes positioned in common by both build 6 and build 7 of the pig genome sequence, dark blue are genes positioned differently by build 6, orange are genes positioned by version 6 and human orthologues, green are genes that could not be positioned by human orthologues or build 6. Red bars denote confidence intervals for QTL associated with glucose, cortisol and ACTH from analyses by Desautes et al., 2002.

The localization of the twelve differentially expressed genes on the pig genome has been realised by two different methods. First, the genes have been aligned to the human genome then mapped on the pig chromosomes by synteny using the comparative map [7]. Second, the genes have been directly aligned to the draft pig genome sequence. The different results are compared in Table 1. The Eif1b, Crem, A2m and Rnf2 genes have been mapped on chromosomes 13, 10, 5 and 1, respectively by both methods. Cited1 has been localized on the pig chromosome X and the human chromosome X. The Pig RH map – human comparative map is not available for X/Y chromosomes [7]. Nevertheless, the comparative tool from the Rat Genome Database [15] showed similarities between the X chromosomes from human, rat and mouse genomes, thus it can be hypothesized that the X chromosomes from the human and the pig genomes are conserved. The Ckb gene has been mapped on chromosome X by alignment against the pig genome and on chromosome 7 by alignment on a human genome region that in syntenic with porcine chromosome 7. Anpep and Cd83 have only been mapped on chromosome 7 by alignment against pig genome. The Gadd45b, Ldlr, Star and Acox1 genes have only been mapped on chromosomes 2, 2, 15 and 12, respectively by synteny after alignment against human genome. The comparative map [7] showed syntenic blocks. According to the "synteny blocks" definition from Pevzner and Tesler [16], the Anpep, Gadd45b, Ckb, Ldlr, Star, Acox1 and Cd83 genes should be mapped on dissimilar regions. The consensus location of all 12 genes is highlighted in Figure 2, including the 4 genes that could not initially be mapped to the porcine sequence but were successfully mapped to version 7 of the draft genome (Roslin).

**Table 1:** Location of 12 most significant genes using human genome sequence, porcine syntenic regions, and draft porcine genome assembly (version 6 and 7, respectively)

name	Human chr.	Human genome position	Pig chr. by synteny	position	Pig chr by blast (v6)	position	Pig chr by blast (v7)	position
Anpep	15	88,129,550..88,135,197	/	/	7	59,359,968..59,347,462	7	60,228,156..60,227,885
Eif1b	3	40,326,322..40,328,830	13	40–45 cM	13	10,798,899..10,801,421	13	16,118,664..16,119,048
Gadd45b	19	2,427,260..2,428,668	2	60–65 cM	/	/	17	13,001,040..13.001,021
Crem	10	35,535,828..35,540,700	10	94–101 cM	10	25,761,249..25,770,177	10	35,728,178..35,728,699
A2m	12	9,116,279..9,126,679	5	90–95 cM	5	39,159,061..39,164,309	5	46,924,367..46,924,494
Ckb	14	103,055,770..103,056,563	7	155–160 cM	X	32,225,303..32,224,867	X	41,876,923..41,876,617
Ldlr	19	11,091,910..11,092,048	2	63–67 cM	/	/	1	65,742,487..65,742,468
Star	8	38,120,950..38,127,503	15	58–63 cM	/	/	8	56,056,948..56,056,928
Acox1	17	71,454,314..71,457,157	12	103–107 cM	/	/	12	1,911,202..1,911,412
Rnf2	9	6,658,602..6,659,714	1	85–90 cM	1	137,816,861..137,816,168	1	155,801,1077..155,800,384
Cited1	X	71,438,228..71,438,760	X human	/	X	25,125,348..25,125,009	X	33,011,256..33,010,917
Cd83	/	/	/	/	7	9,859,712..9,859,943	7	10,204,325..10,204,556

Each of the genes that were mapped on the pig genome has been co-localized with published QTL from pigQTLdb. Eif1b gene has been co-localized with 15 QTL, Ldlr gene with 29 QTL, Crem gene with 10 QTL, Gadd45b gene with 29 QTL, A2m gene with 13 QTL, Ckb gene with 7 QTL, Star gene with 19 QTL, Acox1 gene with 5 QTL and Rnf2 gene with 22 QTL. Only the results for the Ldlr gene are detailed here (Table 2). The results for the other genes are in the Additional file 2. The Ldlr gene has been located on chromosome 2 from 63 to 67 cM. 29 QTL from 14 publications for 28 different traits have been co-localised with Ldlr gene. The "pH for *Longissmus dorsi*" trait has been associated to two loci (QTL_id 791 and 2976). Each locus corresponded to one publication. The QTL 781 has been reported by Su et al. [17] from an F2 cross between Large White and Meishan pigs. This QTL was located from 59.5 to 72.4 cM on SSC2. The QTL 2976 has been studied by Rohrer *et al. *[18] in a Duroc × Landrace F2 population. It has been mapped to an interval from 63.2 to 74.8 cM.

**Table 2:** QTL in the Ldlr gene region of pig chromosome 2.

QTL_ID	QTL_symbol	Trait_name	Position_cM	range_cM	Publication
307	BFT	Average backfat	44,8	42–64,3	Rattink *et al*. [22]
3107	FP	fat ratio (percentage)	49,8	42,3–64,8	Stratil *et al*. [23]
275	vnum	Vertebra number	53	42–59,5	Wada *et al*. [24]
669	Lmdepth	Loin depth at the last ribs	54	45–81	Varona *et al*. [25]
3116	MP	Melting Point	54	53,5–59,5	Nii *et al*. [26]
809	LEANWT	Lean mass (weight)	56	42–70	Geldermann *et al*. [27]
907	ABF	abdominal fat	59,3	57,4–66,1	Lee *et al*. [28]
3224	JUICES	subjective juiciness score	59,5	60,6–59,5	Edwards *et al*. [29]
748	LUMBF	backfat between the last 3th and 4th lumbar	60		Qu *et al*. [30]
749	LEANP	Lean meat percentage	60		Qu *et al*. [30]
2973	shefor	shear force	61	60,6–63,2	Rohrer *et al*. [18]
2974	SJCS	subjective chew score	62	60,6–63,2	Rohrer *et al*. [18]
911	liverwt	Liver weight	62,4	57,4–66,1	Lee *et al*. [28]
2796	DiaMF	Diameter of Muscle Fiber	63	62,9–85,9	Wimmers *et al*. [31]
2797	DiaMF	Diameter of Muscle Fiber	63	62,9–85,9	Wimmers *et al*. [31]
906	pH	pH 24 hours post mortem (loin)	63,6	57,4–66,1	Lee *et al*. [28]
912	efatsho	External fat on shoulder	64,2	57,4–66,1	Lee *et al*. [28]
3039	ihern	Inguinal Hernia	64,3		Grindflek *et al*. [32]
908	BFW	backfat weight	64,9	57,4–66,1	Lee *et al*. [28]
747	34ribBF	backfat between the last 3th and 4th rib	65		Qu *et al*. [30]
3225	off-flavor	subjective off-flavor score	65,1	85,1–60,6	Edwards *et al*. [29]
913	LEA	loin eye area	65,5	57,4–66,1	Lee *et al*. [28]
2798	DiaMF	Diameter of Muscle Fiber	66	62,9–85,9	Wimmers *et al*. [31]
781	pH	pH for Longissmus Dorsi	67	59,5–72,4	Su *et al*. [17]
2975	MMP	muscle moisture percentage	67	63,2–74,8	Rohrer *et al*. [18]
2976	pH	pH for *Longissmus Dorsi*	67	63,2–74,8	Rohrer *et al*. [18]
670	loineyea	Loin muscle area	68	61–80	Varona *et al*. [25]
82	mcolorl	Hormel loin Minolta	72,4	55–77,9	Malek *et al*. [33]
88	whcap	water holding capacity	74,8	55–74,8	Malek *et al*. [33]

QTL_id corresponds to the unique identifier in PigQTLdb. Position_cM corresponds to the peak QTL position and range_cM to the interval QTL position. Chr: chromosome.

### Analysis of all the differentially expressed genes and QTL for stress responses (INRA)

From the 237 sequences in BIOMART, 214 were systematically localized on the human genome and 23 sequences could not be mapped. The differentially expressed genes identified as being co-localized with a QTL for the corticotrope axis are presented in supplementary Table S1 of Additional file 1.

One question is to know whether co-localization between regulated genes and a QTL is significant. The probability of finding the observed genes and a QTL in the same genomic region by random chance could be estimated. The complete pig genome is about 2255 cM for the 18 autosomes. Using R software , the exact binomial test is about 4–35% for the co-localization by chance of a subset of regulated genes among the 211 regulated genes. The probability is high (35%) for the co-localization of three regulated genes with a QTL region of 30 cM (SSC1) by chance. However, in another example the QTL at 17 cM on SSC5 shows an interval of only 2 cM in which we find 7 differentially expressed genes. The probability should be very low to find seven differentially expressed genes by chance in this region. But the comparative mapping is imprecise for this region and a region of 50 cM should be taken into account. Nevertheless, the probability to find by chance seven regulated genes and a QTL in the same region of 50 cM is less than 4%.

### Mapping all differentially expressed genes to the draft genome sequence (AARHUS, ROSLIN)

Using version 6 of the draft genome (AARHUS), 33 genes (13.8%) could be mapped to the porcine genome. Those genes which were mapped were spread over the different chromosomes and did not cluster on a specific chromosome or on a specific chromosomal region. Using version 7 of the draft sequence, the blast search yielded 8000 hits and many genes mapped to multiple chromosomes. The top hit for each gene was used as the most likely position using the bit score and e-values. The Blast output is available from SR on request. Figure 2 shows the distribution of the genes across the chromosomes. Figure 2 also indicates the gene locations identified by INRA and AARHUS as well as any discrepancies between the different mappings. The QTL from Désautés *et al. *(2002) [19] are also represented in Figure 2.

### Differentially expressed genes in the QTL regions (ROSLIN)

Visual inspection of Figure 2 illustrates that there is no obvious enrichment of differentially expressed genes in the QTL regions. In supplementary Tables S2 and S3 of Additional file 1 the differentially expressed genes mapping to the QTL regions for chromosomes 1 and 7, respectively, are listed. The analyses of genes in QTL regions show many discrepancies. For example, some genes have a blast hit within the QTL region but the blast score is highest either elsewhere in the genome or on the chromosome: STAR has a blast hit within the QTL region on chromosome 1 but the best hit from the blast search is on SSC8. There is a STAR-like gene found under the QTL peak for chromosome 1. Similarly Notch 4 maps to the QTL region on SSC7 but the best hit again is outside the QTL region.

Differentially expressed genes close to the QTL region on chromosome 1 include Thioredoxin (Txn) associated with a glucocorticoid receptor and insulin, and fast growth in pigs [20]. The 95 genes identified under the QTL peak included TXN, transforming growth factor beta receptor (TGFBR), 15 genes annotated as 'similar to olfactory receptors', and a steroidogenic regulator or STAR-like domain.

Differentially expressed genes on chromosome 7 mapping to the QTL region include Anpep associated with glutathione metabolism and psychological disorders in humans, and Cntf associated with body mass, obesity and psychiatric disorders in humans. Anpep is upregulated in the Meishan, Anpep and Cntf are down regulated by ACTH.

For the 130 genes mapping under the QTL peak on chromosome 7, most were not annotated. However, 20 loci were similar to olfactory receptors, 1 to thyroid hormone receptor, 5 to glutathione S-transferase including metabolism of xenobiotics and drugs by cytochrome P450, 2 to goosecoid receptors and 1 to cytochrome P450. The Ephx gene on chromosome 10 is also associated with cytochrome P450. The steroid metabolism pathway contains many of the cytochrome P450 substrates.

Using all of the genes under the QTL peaks, we looked for pathways they might be involved in. For chromosome 1 we found 95 genes in the QTL region with KEGG identifiers of which 2 mapped to 13 different pathways. For chromosome 7 we identified 130 genes in the QTL region of which only 2 mapped to 4 pathways. It is likely that these results are heavily biased by annotation and therefore of limited use. The next step would be to use homologues to examine the putative pathways. Definition of a gene universe would also be required in order to test sets of genes for enrichment (see companion paper from pathway analyses group [21]). This illustrates that one of the main objectives, comparison of differentially expressed genes and QTL regions at the pathway level, is very challenging for non-model species.

### Some observations

The integration of QTL data and microarray data for a non-model species like pigs is extra challenging because of the limited annotation and lack of a complete draft genome sequence. While using the most recent draft of the porcine genome offers the most direct route to locate differentially expressed genes and markers flanking QTL region, it is only a pre-Ensembl draft sequence. This means that the assembly is still incomplete and likely to contain a large number of errors. Therefore, the location of genes or markers using this draft sequence is by no means the gold standard against which other, comparative, approaches should be compared. Even for a limited number of genes, the different routes of mapping these genes can cause discrepancies (Table 1 and Figure 2). A good example is the Rnf2, which maps to chromosome 1, using the BLAST searches of the porcine genome. Further study has now shown the gene on SSC1 is a pseudo gene and the actual gene location is on SSC9 (Laurence Liaubet, unpublished data). The comparative mapping approach that can be helpful in locating genes and crucial for annotation and pathway analyses suffers from considerable drop-out rates for each step of the analyses. E.g. when starting with 130 genes in a QTL region on SSC7, only a handful could eventually be mapped to functional pathways. The limited level of annotation also affects analyses that studies overlap between enriched pathways and QTL regions. If only a proportion of the genes can be attributed to pathways, any test for functional enrichment becomes very unreliable. This is discussed in more detail in one of the companion papers [21].

In spite of all the reservations, this case study illustrates that different routes can be taken to link gene expression results with QTL results. It was illustrated that the ideas easily extrapolate from in-house experiments to public domain data. In theory, one could do discovery science based on public domain gene expression results and QTL databases. These would be expected to generate novel hypotheses about candidate genes under the QTL that could be tested further. In the current example the level of genome information is still too sketchy to identify clear positional candidate genes for the QTL. However, it is expected that the same data could be revisited in some years time and yield more fruitful results. Because in EADGENE and SABRE we are working with outbred livestock species, it was decided to use the type of data that is representative of our work. The analyses presented here provide a starting point for integration of microarray and QTL studies that is portable to other non-model species.

## Conclusion

The study lays out a protocol for the examination of QTL and microarray data and suggests ways in which the data can be combined to provide more information.

The different approaches illustrated both the potential and limitation of these approaches. The lack of a reliable genome sequence and poor annotation of the draft assembly were the main challenges that hampered integration of results. Nonetheless the results demonstrated that much of the integration can be achieved using basic bioinformatics tools like PERL scripts and public databases.

## Competing interests

The authors declare that they have no competing interests.

## Authors' contributions

SR, VJ, LL, BB and HH analysed the data. MSC, PM and DJK coordinated the analyses. All authors contributed to the manuscript. PM coordinated the experiments that provided the QTL and microarray data for the analyses presented in this manuscript.

## Supplementary Material

Additional file 1 Supplementary Tables S1, S2 and S3 Click here for file

Additional file 2 Public domain QTL that co-locate with the top 12 differentially expressed genes. Click here for file

Additional file 3 A copy of the bash scripts used by ROSLIN Click here for file
