# Supplementary material for: Using microarrays to identify positional candidate genes for QTL: the case study of ACTH response in pigs
Source: BMC Proc. 2009 Jul 16;3(Suppl 4):S14. doi: 10.1186/1753-6561-3-S4-S14 (PMC2712744; doi:10.1186/1753-6561-3-S4-S14)
Supplement: Additional file 1 — Supplementary Tables S1, S2 and S3 [file 1753-6561-3-S4-S14-S1.doc]

**Supplementary Tables**

**Table S1. Identification of genes localised in the QTL for the corticotrope axis**

| SSC | QTL | cM | HSA | Mb | HSA | Position (bases) | Accession Nb | Gene name | Description |
| --- | --- | --- | --- | --- | --- | --- | --- | --- | --- |
| 1 | ACTH2, dACTH | 94-125 | 9 | 35-37 | 9 | 76893279 | BX925447 | OSTF1 | osteoclast stimulating factor (OSTF1) |
|  | GLU2, LOC |  | 9 | 74-118 | 9 | 81196014 | BX921605 | CHCHD9 | coiled-coil-helix-coiled-coil-helix domain containing 9 |
|  |  |  |  |  | 9 | 112045912 | BX918976 | TXN | thioredoxin |
| 3 | GLU1 | 110 | 2 | 14-16 | none |  |  |  |  |
| 5 | GLU2 | 17 | 12 | 54-107 | 12 | 53260992 | BX671712 | PPP1R1A | protein phosphatase 1, regulatory (inhibitor) subunit 1A |
|  |  |  | or 22 | or 31-42 | 12 | 55318230 | BX668204 | ATP5B | ATP synthase, H+ transporting, mitochondrial F1 complex, beta |
|  |  |  |  |  | 12 | 107479369 | BX667428 | ISCU | iron-sulfur cluster scaffold homolog |
|  |  |  |  |  | 22 | 31526802 | BX918784 | CHRNA3 | cholinergic receptor, nicotinic, alpha 3 |
|  |  |  |  |  | 22 | 35236844 | BX926369 | EIF3D | Eukaryotic translation initiation factor 3 subunit |
|  |  |  |  |  | 22 | 37460689 | BX667686 | UNC84B | unc-84 homolog B |
|  |  |  |  |  | 22 | 41892572 | BX676197 | TTLL12 | tubulin tyrosine ligase-like family, member 12 |
| 5 | dGLU | 123 | 12 | 97-102 | none |  |  |  |  |
| 7 | CORT1,2, dCORT | 104-156 | 14 | 74-100 | 14 | 76561498 | BI359937 | C14orf4 | Homo sapiens chromosome 14 open reading frame 4 |
| 8 | CORT2 | 102-108 | 4 | 96-100 | none |  |  |  |  |
| 11 | CORT1 | 5 | 13 | 20-34 | none |  |  |  |  |
| 17 | ACTH2 | 54 | 20 | 1-4, 14-25 | 20 | 1297623 | BX676563 | FKBP-12 | FK506-binding protein 1A |
| 18 | CORT1, dCORT | 0-3 | 7 | 140-156 | none |  |  |  |  |

**Table S2. Identification of differentially genes localised in the QTL region on chromosome 1.**

| Query | identity | Alignment  length | Mis  match | E  value | Bit  score | gene  symbol | gene  name | gene function |
| --- | --- | --- | --- | --- | --- | --- | --- | --- |
| BX926397 | 91.59 | 214 | 14 | 5E-67 | 260 | Hik1 | potassium channel, subfamily K, member 13 | Unknown |
| BX673517 | 88.09 | 705 | 71 | 0 | 700 | Rnf2 | ring finger protein 2 | Transcription regulation |
| BX918976 | 84.32 | 185 | 22 | 3E-25 | 121 | Txn | thioredoxin | Signal Transduction  Electron transport |
| CR939969 | 93.94 | 33 | 2 | 0.0009 | 50.1 | unknown | unknown | Unknown |
| BX919206 | 89.74 | 78 | 6 | 2E-14 | 85.7 | unknown | unknown | Unknown |
| BX925141 | 88.93 | 307 | 34 | 7E-91 | 339 | Rpl36a | ribosomal protein L36a | Ribosome Constituent |

**Table S3. Identification of differentially expressed genes localised in the QTL region on chromosome 7.**

| Query | identity | Alignment  length | Mis  match | E  value | Bit  score | gene  symbol | gene  name | gene function |
| --- | --- | --- | --- | --- | --- | --- | --- | --- |
| BX915371 | 89.66 | 116 | 12 | 2.00E-29 | 135 | Nr2f1 | nuclear receptor subfamily 2,  group F, member 1 | Transcription  regulation |
| BX922805 | 100 | 23 | 0 | 0.018 | 46.1 | Cntf | ciliary neurotrophic factor | Signal transduction |
| BI359937 | 99.45 | 546 | 2 | 0 | 1051 | C14orf4 | chromosome 14  open reading frame 4 | unknown |
| BX919206 | 92.06 | 63 | 5 | 2.00E-14 | 85.7 | unknown | unknown | unknown |
| BX670249 | 93.94 | 33 | 2 | 0.001 | 50.1 | Notch4 | Notch homolog 4  (Drosophila) | Ion binding  /heterodimerization  /receptor activity |
| BX676718 | 98.66 | 298 | 3 | 7.00E-155 | 551 | C14orf2 | chromosome 14  open reading frame 2 | unknown |
