# Supplementary material for: Using microarrays to identify positional candidate genes for QTL: the case study of ACTH response in pigs
Source: BMC Proc. 2009 Jul 16;3(Suppl 4):S14. doi: 10.1186/1753-6561-3-S4-S14 (PMC2712744; doi:10.1186/1753-6561-3-S4-S14)
Supplement: Additional file 2 — Public domain QTL that co-locate with the top 12 differentially expressed genes. [file 1753-6561-3-S4-S14-S2.doc]

**Supplementary file 2: QTL from published studies that co-locate with the 12 top genes (INRA)**

The QTL_ID refers to the identifier of a QTL in PigQTLdb <http://www.animalgenome.org/QTLdb/pig.html>. The range_cM described the confidence interval of the QTL (if available)

1: QTLs co-localized with Gadd45b. Gadd45b has been mapped by synteny on pig linkage group 2 (60-65 cM).

| QTL_ID | QTL_symbol | Trait_name | Chromosome | Position_cM | range_cM |
| --- | --- | --- | --- | --- | --- |
| 307 | BFT | Average backfat | 2 | 44,8 | 42-64,3 |
| 3107 | FP | fat ratio (percentage) | 2 | 49,8 | 42,3-64,8 |
| 275 | vnum | Vertebra number | 2 | 53 | 42-59,5 |
| 669 | Lmdepth | Loin depth at the last ribs | 2 | 54 | 45-81 |
| 3116 | MP | Melting Point | 2 | 54 | 53,5-59,5 |
| 809 | LEANWT | Lean mass (weight) | 2 | 56 | 42-70 |
| 907 | ABF | abdominal fat | 2 | 59,3 | 57,4-66,1 |
| 3224 | JUICES | subjective juiciness score | 2 | 59,5 | 60,6-59,5 |
| 748 | LUMBF | backfat between the last 3th and 4th lumbar | 2 | 60 |  |
| 749 | LEANP | Lean meat percentage | 2 | 60 |  |
| 2973 | shefor | shear force | 2 | 61 | 60,6-63,2 |
| 2974 | SJCS | subjective chew score | 2 | 62 | 60,6-63,2 |
| 911 | liverwt | Liver weight | 2 | 62,4 | 57,4-66,1 |
| 2796 | DiaMF | Diameter of Muscle Fiber | 2 | 63 | 62,9-85,9 |
| 2797 | DiaMF | Diameter of Muscle Fiber | 2 | 63 | 62,9-85,9 |
| 906 | pH | pH 24 hours post mortem (loin) | 2 | 63,6 | 57,4-66,1 |
| 912 | efatsho | External fat on shoulder | 2 | 64,2 | 57,4-66,1 |
| 3039 | ihern | Inguinal Hernia | 2 | 64,3 |  |
| 908 | BFW | backfat weight | 2 | 64,9 | 57,4-66,1 |
| 747 | 34ribBF | backfat between the last 3th and 4th rib | 2 | 65 |  |
| 3225 | off-flavor | subjective off-flavor score | 2 | 65,1 | 85,1-60,6 |
| 913 | LEA | loin eye area | 2 | 65,5 | 57,4-66,1 |
| 2798 | DiaMF | Diameter of Muscle Fiber | 2 | 66 | 62,9-85,9 |
| 781 | pH | pH for Longissmus Dorsi | 2 | 67 | 59,5-72,4 |
| 2975 | MMP | muscle moisture percentage | 2 | 67 | 63,2-74,8 |
| 2976 | pH | pH for Longissmus Dorsi | 2 | 67 | 63,2-74,8 |
| 670 | loineyea | Loin muscle area | 2 | 68 | 61-80 |
| 82 | mcolorl | Hormel loin Minolta | 2 | 72,4 | 55-77,9 |
| 88 | whcap | water holding capacity | 2 | 74,8 | 55-74,8 |

2: QTLs co-localized with Ckb. Ckb has been mapped by synteny on pig linkage group 7 (155-160 cM).

| QTL_ID | QTL_symbol | Trait_name | Chromosome | Position_cm | range_cm |
| --- | --- | --- | --- | --- | --- |
| 482 | CORT1 | Basla cortisol levels | 7 | 134,9 | 104,4-156 |
| 483 | CORT2 | Post stress cortisol levels | 7 | 134,9 | 104,4-156 |
| 3653 | BWL | body weight linear | 7 | 149,8 | 156-134,9 |
| 484 | dCORT | Cortisol variations | 7 | 156 | 134,9-156 |
| 729 | IFR | Internal fat percentage | 7 | 157 |  |
| 777 | crcl | Carcass length | 7 | 157 |  |
| 2812 | DiaMF | Diameter of Muscle Fiber | 7 | 168 | 142,6-172,8 |

3: QTLs co-localized with A2m. A2m has been mapped by synteny on pig linkage group 5 (90-95 cM).

| QTL_ID | QTL_symbol | Trait_name | Chromosome | Position_cm | range_cm |
| --- | --- | --- | --- | --- | --- |
| 351 | BFT | Average backfat (ultasound) | 5 | 93 | 88,2-118,7 |
| 3060 | hamwt | Ham weight | 5 | 93 | 88,2-108 |
| 3044 | ihern | Inguinal Hernia | 5 | 95,5 |  |
| 342 | LGR | Life growth rate | 5 | 96 | 88,2-118,7 |
| 3307 | 24CARTEMP | 24 h carcass temperature | 5 | 96,9 | 9,7-103,3 |
| 3644 | PCL | empty body protein linear | 5 | 99 | 108-88,2 |
| 3114 | SFA | Total Saturated Fatty Acids | 5 | 102,5 | 95,5-109,4 |
| 987 | con24hr | conductivity 24 hours post mortem | 5 | 106,3 | 88,2-107 |
| 3113 | FA-UI | Unsaturated index | 5 | 107,1 | 95,5-109,4 |
| 3128 | FA-UI | Unsaturated index | 5 | 108,5 | 95,5-109,4 |
| 101 | pH | lab loin pH | 5 | 109,4 | 79,4-130,3 |
| 413 | bellywt | Belly weight | 5 | 110 | 90-127 |
| 383 | LHP | Loin and ham percentage in carcass | 5 | 118 | 89-127 |

4: QTLs co-localized with Crem. Crem has been mapped by synteny on pig linkage group 10 (94-101 cM).

| QTL_ID | QTL_symbol | Trait_name | Chromosome | Position_cm | range_cm |
| --- | --- | --- | --- | --- | --- |
| 2928 | TNUM | Number of nipples | 10 | 83 | 79,4-108 |
| 594 | TNUM | Number of nipples | 10 | 86,3 | 67,5-101 |
| 2952 | LRIBFD | backfat depth at last rib | 10 | 86,3 | 86,3-108 |
| 518 | OVRATE | Ovulation rate | 10 | 89 | 44-118 |
| 1281 | efatham | External fat on ham | 10 | 93 | 91,0-94,5 |
| 280 | ADG | average daily gain (4-13 weeks) | 10 | 99 | 94,5-101 |
| 646 | FSH | Plasma FSH concentration | 10 | 101 | 101-108 |
| 1283 | STWt | Musculus semitendinosus (ST) weight | 10 | 101 | 96,0-108,0 |
| 1282 | SMAWt | Musculus semimembranosus et adductor (SMA) weight | 10 | 105 | 101,0-108,0 |
| 1107 | TNUM | Number of nipples | 10 | 113 | 101-128 |

5: QTLs co-localized with Eif1b. Eif1b has been mapped by synteny on pig linkage group 13 (40-45 cM).

| QTL_ID | QTL_symbol | Trait_name | Chromosome | Position_cm | range_cm |
| --- | --- | --- | --- | --- | --- |
| 3075 | hamwt | Ham weight | 13 | 24 | 22,6-43,1 |
| 3003 | juices | subjective juiciness score | 13 | 31 | 22,6-43,1 |
| 3073 | pH | pH 24 hours post mortem (loin) | 13 | 36 | 22,6-43,1 |
| 3078 | hmeatwt | Ham Meat Weight | 13 | 37 | 22,6-43,1 |
| 347 | LGR | Life growth rate | 13 | 42 | 27,9-62,2 |
| 113 | whcap | water holding capacity | 13 | 43 |  |
| 328 | EGR | Early growth rate | 13 | 43 | 27,9-62,2 |
| 1132 | BFW | backfat weight | 13 | 43,1 | 27,9-62,2 |
| 1147 | con45min | conductivity 45 minutes post mortem | 13 | 43,1 | 27,9-53 |
| 2900 | ADG | average daily gain | 13 | 43,1 |  |
| 2922 | BFT | backfat (average) thickness | 13 | 43,1 |  |
| 336 | TGR | Test growth rate | 13 | 44 | 27,9-62,2 |
| 1141 | FP | fat ratio (percentage) | 13 | 45,6 | 43,1-53 |
| 1138 | con24hr | conductivity 24 hours post mortem | 13 | 51,4 | 43,1-53 |
| 1131 | con45min | conductivity 45 minutes post mortem | 13 | 59,8 | 43,1-62,2 |

6: QTLs co-localized with Acox1. Acox1 has been mapped by synteny on pig linkage group 12 (103-107 cM).

| QTL_ID | QTL_symbol | Trait_name | Chromosome | Position_cm | range_cm |
| --- | --- | --- | --- | --- | --- |
| 3085 | LEA | loin eye area | 12 | 94 | 63 -108 |
| 157 | imm | Stress-induced alterations in mitogen induced IL-2 activity | 12 | 102 | 100-104,6 |
| 1125 | HWT | Heart weight | 12 | 108,3 | 80,2-113,1 |
| 1126 | hamwt | Ham weight | 12 | 108,3 | 80,2-113,1 |
| 393 | hamwt | Ham weight | 12 | 110 | 93-113 |

7: QTLs co-localized with Star. Star has been mapped by synteny on pig linkage group 15 (58-63 cM).

| QTL_ID | QTL_symbol | Trait_name | Chromosome | Position_cm | range_cm |
| --- | --- | --- | --- | --- | --- |
| 1243 | dressing | Dressing | 15 | 35 | 34,6-57,09 |
| 2964 | pH | pH 48 hours post mortem (loin) | 15 | 53 | 50,7-67,5 |
| 3658 | LEAI | Loin Eye Area Intercept | 15 | 53,1 | 65,1-34,6 |
| 123 | pH | lab loin pH | 15 | 56 | 39,8-73,1 |
| 2829 | TNoF | Total Number of Fibers | 15 | 56 | 36,6-85,1 |
| 2830 | ProMF | Proportion Of Muscle Fiber | 15 | 57 | 36,6-85,1 |
| 3014 | dripl | drip loss | 15 | 57 | 50,7-70,5 |
| 247 | ADG | average daily gain 70kg | 15 | 62 |  |
| 3015 | shefor | shear force | 15 | 66 | 50,7-70,5 |
| 3291 | PC | Protein Content | 15 | 68,3 | 56,2-87,5 |
| 2831 | DiaMF | Diameter of Muscle Fiber | 15 | 69 | 36,6-85,1 |
| 3311 | MCOLOR | color L | 15 | 72,7 | 51,1-82,4 |
| 519 | OVRATE | Ovulation rate | 15 | 79 | 53-101 |
| 3294 | tend | subjective tenderness score | 15 | 79,9 | 44,0-115,9 |
| 3295 | TEND | tenderness score | 15 | 79,9 | 43,0-107,2 |
| 2832 | DiaMF | Diameter of Muscle Fiber | 15 | 81 | 36,6-85,1 |
| 2833 | DiaMF | Diameter of Muscle Fiber | 15 | 84 | 36,6-85,1 |
| 122 | pH | Hormel loin pH | 15 | 95 | 56-113,1 |
| 293 | pH | 24 hr loin pH | 15 | 95 | 56-119,9 |

8: QTLs co-localized with Rnf2. Rnf2 has been mapped by synteny on pig linkage group 1 (85-90 cM).

| QTL_ID | QTL_symbol | Trait_name | Chromosome | Position_cm | range_cm |
| --- | --- | --- | --- | --- | --- |
| 797 | BFT | backfat (average) thickness | 1 | 80 | 75-85 |
| 44 | LRIBBF | Last-rib back fat | 1 | 80,5 | 73-112,5 |
| 3171 | MC4R | backfat weight | 1 | 80,5 | 61,3-100,8 |
| 651 | BFTR | Back fat at rump. | 1 | 81 | 55,8-93,9 |
| 835 | dressing | dressing percentage | 1 | 81,94 | 81-94,3 |
| 2845 | bellywt | belly weight | 1 | 83 | 58,5-86,2 |
| 850 | LRIBFD | backfat depth at last rib | 1 | 84,2 | 81-94,3 |
| 867 | 34ribBF | backfat between 3th and 4th rib | 1 | 84,2 | 81-94,3 |
| 840 | efatsho | External fat on shoulder | 1 | 84,55 | 81-94,3 |
| 842 | FATCP | Fat-cut percentage | 1 | 84,74 | 81-94,3 |
| 45 | LUMBF | Last lumbar backfat | 1 | 86,2 | 67,6-100,8 |
| 2844 | BFT | backfat (average) thickness | 1 | 87 | 86,2-100,8 |
| 864 | BFT | backfat (average) thickness | 1 | 87,3 | 81-94,3 |
| 848 | pH | pH 24 hours post mortem (loin) | 1 | 87,4 | 81-94,3 |
| 855 | FATCP | Fat-cut percentage | 1 | 88,3 | 81-94,3 |
| 686 | FA-C18:3 | linoleic acis | 1 | 90,7 | 80,5-102,9 |
| 432 | headwt | Head weight | 1 | 92 | 80-105 |
| 1265 | SHOFTWT | Shoulder fat weight | 1 | 92 | 86,2-93,9 |
| 2794 | PoMF | Proportion Of Muscle Fiber | 1 | 93 | 76,1-137,7 |
| 2848 | PAR | Protein Accretion Rate | 1 | 100 | 86,2-100,8 |
| 2795 | DiaMF | Diameter of Muscle Fiber | 1 | 114 | 76,1-137,7 |
| 1251 | FATCP | Fat / meat ratio | 1 | 116 | 84,3-122,6 |
