# Supplementary material for: Using microarrays to identify positional candidate genes for QTL: the case study of ACTH response in pigs
Source: BMC Proc. 2009 Jul 16;3(Suppl 4):S14. doi: 10.1186/1753-6561-3-S4-S14 (PMC2712744; doi:10.1186/1753-6561-3-S4-S14)
Supplement: Additional file 3 — A copy of the bash scripts used by ROSLIN [file 1753-6561-3-S4-S14-S3.doc]

**A copy of the scripts used by ROSLIN**

The following script takes a list of accession numbers and uses then to retrieve fasta sequence files for each gene using the emboss software package. The sequences are then blasted against the latest version of the pig genome (7) which was downloaded from the Sanger Institute website. Before using blastall to position the genes, the pig genome was converted to a searchable database using formatdb.

### copy data table and get accession numbers#####

cat Copy\ of\ Supplemental_data_Table1.txt | awk 'BEGIN {FS="\t"}; NR>8 {print $4}' | sed "s/\//\n/" | grep -v "^$" | grep -v "NO_DATA" > accessions.txt

### same thing but now data table is named hazdat####

cat hazdat.txt | awk 'BEGIN {FS="\t"}; NR>8 {print $4}' | sed "s/\//\n/" | grep -v "^$" | grep -v "NO_DATA" > accessions.txt

### take the accession numbers and get fasta sequence files####

for seq in `cat accesssions.txt`; do seqret genbank:${seq} -outseq ${seq}.fasta; done

### same thing using list command from emboss ###

seqret list:accessions.txt -outseq fastseqall

### move to folder where sanger sequence for version & pig sequence is saved and unzipped ###

cd Sus_scrofa.Sscrofa7.47.dna.chromosome.fa

### give it a snappier title ###

mv Sus_scrofa.Sscrofa7.47.dna.chromosome.fa sscrofa.fa

### convert genome sequence to searchable database using formatdb

formatdb -i sscrofa.fa -p F

more formatdb.log

### check for appropriate database files n for nucleotide p for protein ###

more sscrofa.fa.nhr

more sscrofa.fa.nin

more sscrofa.fa.nsq

### use blastall (blastn for nucleotide search) using -F T filter is true, -W 20 - word length of 20, -b 2, restricted output, -m 9 XML format for output, ###

blastall -p blastn -d sscrofa.fa -F T -W 20 -b 2 -m 9 -i allseq.fasta -o allseqblast.out

Fields: Query id, Subject id, % identity, alignment length, mismatches, gap openings, q. start, q. end, s. start, s. end, e-value, bit score

#### same for markers ####

seqret list:markaccess.txt -outseq markout

### using word length of 20 and XML format ###

blastall -p blastn -d sscrofa.fa -W 20 -m 9 -i markout -o markblast3.out

### using word length of 20, XML format and setting threshold for e-value to 0.001 ###

blastall -p blastn -d sscrofa.fa -W 20 -m 9 -e 1000 -i markout -o markblast3.out
